# Normoglycemic Diabetes—A New Disease Concept Complementary to Type 2 Diabetes Mellitus Based on Early Beta-Cell Dysfunction

**DOI:** 10.3390/jpm16100522

**Published:** 2026-10-09

**Authors:** Julia Jantz, Petra Wiechel, Uwe Nixdorff, Andreas Pfützner

**Affiliations:** 1 Pfützner Science & Health Institute, 55128 Mainz, Germany; julia.jantz@fpuetzner-mainz.com; 2 Swiss Mountain Clinic, CH-6540 Castaneda, Switzerland; petra.wiechel@swissmountainclinic.com; 3 European Prevention Center, 40235 Duesseldorf, Germany; nixdorff@epccheckup.de; 4 Department of Biotechnology, University for Applied Sciences, 55411 Bingen, Germany

**Keywords:** normoglycemic diabetes, β-cell dysfunction, functional β-cell mass, intact proinsulin, proinsulin-to-insulin ratio, type 2 diabetes, preclinical diabetes, precision medicine

## Abstract

**Background:** The conventional definition of diabetes mellitus is based on the presence of hyperglycemia. However, growing evidence indicates that key pathophysiological processes—particularly β-cell dysfunction—occur well before any measurable increase in blood glucose. These early changes remain undetected by standard diagnostics but are clinically significant. **Objective:** This work introduces the proposed concept of normoglycemic diabetes, defined as a pathophysiological state in which conventional glucose criteria remain normal while measurable evidence of early β-cell dysfunction is already present. Particular emphasis is placed on intact proinsulin as a marker of impaired proinsulin processing and β-cell secretory stress. **Methods:** A staged model of declining functional β-cell mass is presented, ranging from compensated β-cell stress during normoglycemia to overt secretory failure and dysglycemia, incorporating genetic, epigenetic, and environmental influences. Diagnostic strategies, including extended OGTT assessment and intact proinsulin quantification, and their potential preventive implications are discussed. **Results:** Early impairment of functional β-cell mass can be detectable while fasting glucose, HbA1c, and conventional glucose tolerance criteria remain normal. Intact proinsulin and proinsulin-related ratios may provide information on impaired hormone processing and secretory stress before overt hyperglycemia develops. The proposed concept therefore identifies a biological state that may become a target for earlier, marker-guided prevention. **Conclusions:** Normoglycemic diabetes is proposed as an early pathophysiological disease concept complementary to established glucose-defined categories. It is distinct from diabetes prevention itself: the former describes measurable β-cell pathology during normoglycemia, whereas prevention describes interventions intended to delay or avoid progression to dysglycemia and overt type 2 diabetes. Prospective studies are required before this concept can be considered an established diagnostic entity.

## 1. Introduction

Current clinical diagnostics of diabetes mellitus rely exclusively on the assessment of glucose homeostasis—particularly fasting blood glucose, the oral glucose tolerance test (OGTT), and HbA1c levels [1]. However, these parameters reflect relatively late stages of disease progression, when metabolic regulation is already markedly impaired. In recent years, growing evidence has suggested that relevant pathophysiological processes—especially impaired pancreatic beta-cell function [2,3]—begin long before the onset of hyperglycemia. This early beta-cell dysfunction (BCD) often remains undetected by conventional diagnostic tools but can be identified through specialized laboratory approaches [4,5].

Numerous observations indicate that even in normoglycemic individuals, there may be signs of an inadequate insulin response, disturbed proinsulin processing, and early vascular abnormalities [4,6,7,8]. This discrepancy between normal glucose control and subclinical beta-cell damage suggests that the current classification of diabetes is insufficient to adequately capture the full spectrum of pathophysiological developments. The concept of “normoglycemic diabetes” aims to fill this diagnostic gap and to establish a new framework for the early detection, prevention, and treatment of metabolic disease.

At the center of this new disease model is the identification of beta-cell dysfunction as a primary event that may contribute to the development of diabetes-related complications independently of hyperglycemia. The measurement of intact proinsulin and the proinsulin-to-insulin ratio offer promising diagnostic markers for making incipient beta-cell dysfunction visible already at the normoglycemic stage [9,10].

The aim of this article is to elucidate the physiological basis of beta-cell function, its genetic regulation, and its degeneration across different stages of disease progression. Particular attention is given to intact proinsulin as a central marker and possible mediator of pathophysiological changes. Finally, a clinical picture of normoglycemic diabetes is outlined, and its diagnostic and therapeutic implications are discussed.

Importantly, the proposed concept should not be equated with diabetes prevention. Prevention describes an intervention strategy applied to individuals at increased risk, whereas normoglycemic diabetes is intended to describe an already measurable pathophysiological state: impaired functional β-cell mass despite glucose values that still fulfil conventional criteria for normal glucose tolerance. Recognition of this state may identify a target for prevention, but it is conceptually distinct from the preventive intervention itself. The term is proposed as a hypothesis-generating framework and not as an established diagnostic category.

## 2. Physiology of Beta-Cell Function

The pancreatic beta cell plays a central role in the regulation of glucose homeostasis. It is located within the islets of Langerhans of the endocrine pancreas and is responsible for the synthesis, storage, and release of insulin—the only physiological hormone capable of effectively lowering blood glucose levels. Beta-cell function is tightly linked to the metabolic status of the organism and is regulated in a highly dynamic manner. A precise understanding of its physiological properties is essential for recognizing the early alterations of emerging beta-cell dysfunction, as they underlie the concept of normoglycemic diabetes.

In response to rising blood glucose levels after a meal, insulin secretion occurs in a biphasic pattern. The first phase is characterized by a rapid release, occurring within minutes, of insulin vesicles stored near the cell membrane and represents a crucial early response to the postprandial glucose load [11,12]. The second phase consists of a delayed but sustained secretion that includes both the mobilization of new vesicles and the de novo synthesis of insulin [13,14].

Insulin secretion is physiologically oscillatory, with major secretory pulses occurring approximately every 5–10 min. Coordinated pulsatility contributes to efficient hepatic insulin signalling, triggers and maintains the sensitivity of vascular insulin receptors, and reflects integrated islet function [15,16,17,18]. Disturbance of this temporal organization has been reported early in the evolution of β-cell dysfunction and may occur before overt deterioration of glucose tolerance. However, assessment of pulsatility requires frequent blood sampling and specialized analytical methods; it is therefore discussed here as a physiological manifestation of declining β-cell function, not as a routine diagnostic criterion for normoglycemic diabetes.

The beta cell is equipped with a highly specialized glucose-sensing system, mediated primarily by the glucose transporter GLUT1 in humans and the enzyme glucokinase [19]. The intracellular rise in ATP resulting from glucose metabolism leads to inhibition of ATP-sensitive potassium (K-ATP) channels, depolarization of the cell membrane, and consequent calcium influx—the actual trigger for the exocytosis of insulin vesicles [20]. This signal transduction cascade is susceptible to disruption by chronic overnutrition, lipotoxicity, and inflammatory signals long before hyperglycemia becomes clinically manifest [21,22].

Beta-cell function is closely embedded within the complex cellular architecture of the islets of Langerhans. Interactions with alpha cells (glucagon), delta cells (somatostatin), and pancreatic PP cells modulate insulin secretion through paracrine mechanisms. Neuronal and vascular signals also influence the secretory behavior of beta cells. Disturbed intra-islet communication may lead to an uncoupling of the glucose response without this being immediately reflected in peripheral glucose measurements [23,24].

The beta cell possesses remarkable plasticity. During periods of increased insulin demand—such as obesity, pregnancy, or pubertal growth spurts—it can adapt both its mass and function [25]. These two dimensions should be considered together as functional β-cell mass, i.e., the effective insulin-secretory capacity generated by the amount of β-cell tissue and the functional competence of the individual cells. Functional impairment and structural loss need not occur in parallel: impaired stimulus-secretion coupling, loss of first-phase secretion, and defective proinsulin processing may precede a demonstrable reduction in anatomical β-cell mass. Conversely, loss of β-cell mass increases the workload of the remaining cells and may accelerate functional deterioration. Chronic metabolic overload can therefore reduce functional β-cell mass before hyperglycemia becomes clinically manifest [14,26].

## 3. Genetic Basis of Beta-Cell Dysfunction

The functional capacity of beta cells is largely genetically determined. Studies clearly show that diabetes does not develop in the absence of a genetic predisposition. Individuals without diabetogenic variants—such as those in TCF7L2, MTNR1B, or HNF1A—do not develop diabetes even under unfavourable lifestyle conditions. Conversely, genetic predisposition alone is often sufficient for incipient beta-cell dysfunction to become apparent despite normal glucose levels [27,28,29].

Of particular importance, diabetes is not a self-inflicted disease. Unfavourable diet or physical inactivity may accelerate disease manifestation in genetically predisposed individuals, but they are never the sole cause. This insight helps to destigmatize affected individuals and shifts the focus toward early functional alterations—such as an inadequate insulin response or increased proinsulin secretion—that may occur independently of glucose levels [28,30].

Epigenetic programming, for example through intrauterine factors, as well as microRNAs, may further modulate gene expression and thereby influence individual vulnerability. This genetic and epigenetic disposition may cause beta cells to show an inadequate response even at normal glucose levels [31], with increased proinsulin secretion serving as an early marker. Genetic vulnerability becomes particularly clinically evident under conditions of metabolic stress, such as obesity, psychological stress, or pregnancy [32,33]. Genetically determined beta-cell dysfunction therefore constitutes the foundation of normoglycemic diabetes.

## 4. Stages of Beta-Cell Dysfunction (BCD)

Functional β-cell mass is subject to a dynamic course ranging from compensated adaptation to progressive secretory failure. At the core of the concept of “normoglycemic diabetes” lies the recognition that a decline in effective β-cell secretory capacity can begin while glucose levels are still within the normal range. The model therefore integrates β-cell function and β-cell mass rather than treating them as independent processes. In routine clinical practice, however, functional abnormalities can be measured much more readily than anatomical β-cell mass, and the relative contribution of structural loss cannot usually be determined in an individual patient.

### 4.1. Early Stage: Normoglycemia with Inadequate Insulin Response

Even at the earliest stage, a genetically or epigenetically determined reduction in beta-cell reserve, or an initially impaired signal transduction within the beta cell, may lead to glucose-induced insulin secretion that is delayed, diminished, or inadequately matched to the rise in glucose. Although fasting glucose and HbA1c remain within the normal range at this stage, more detailed analysis—such as an oral glucose tolerance test with insulin profiles—may reveal a blunted or delayed insulin response. A particularly sensitive marker at this stage is an increase in intact proinsulin in peripheral blood, often accompanied by an elevated proinsulin-to-insulin ratio (PI/I). These changes indicate an incipient disturbance in the maturation of insulin secretion and represent an early sign of functional stress in the beta cell [34,35].

### 4.2. Compensated Stage: Hyperinsulinemia with Normoglycemia

In the next stage, the remaining functional β-cell mass is still able to maintain glucose homeostasis through increased insulin release despite rising insulin resistance or metabolic demand. Plasma insulin levels—particularly after glucose stimulation—may be elevated while glucose values remain normal. This compensated state can mask declining β-cell reserve. Loss of first-phase secretion and persistently elevated intact proinsulin may already indicate increasing secretory inefficiency [11,34,35].

### 4.3. Decompensated Stage: Onset of Glucose Dysregulation

With sustained beta-cell overload, decompensation occurs: the secretory capacity is no longer sufficient to maintain glucose levels within the normal range. At this stage, postprandial glucose elevations appear first, followed by impaired fasting glucose and ultimately overt diabetes. It often goes unnoticed, however, that beta-cell dysfunction may precede this stage by many years. Even at this point, the proinsulin-to-insulin ratio remains elevated—often more markedly than in established type 2 diabetes—indicating advanced impairment of proinsulin processing and structural alterations of the beta cells [34,36].

### 4.4. Elevated Intact Proinsulin Is Complementary to the Glucocentric Diabetes Definition

As diabetes-related complications—such as microangiopathic changes (e.g., retinopathy, microalbuminuria, and polyneuropathy), endothelial dysfunction, or metabolic dysfunction-associated steatotic liver disease (MASLD)—may already occur despite normal glucose levels if relevant beta-cell dysfunction is present, the traditional definition of diabetes, centered on hyperglycemia as the key criterion, is insufficient to adequately capture the risk of diabetes-related end-organ damage. Rather, functional parameters of beta-cell health—particularly proinsulin concentration—should be incorporated into risk assessment [37,38].

## 5. Intact Proinsulin: Physiology, Measurement, and Significance as a Marker of β-Cell Dysfunction

Intact proinsulin (iPI) is defined here as the full-length, uncleaved 86-amino-acid precursor of insulin before enzymatic cleavage at the two dibasic processing sites generates insulin and C-peptide. This definition is analytically distinct from “total proinsulin,” which may include intact proinsulin together with partially processed conversion intermediates, depending on assay specificity. The distinction is important because studies using intact, split, and total proinsulin assays are not analytically interchangeable.

Insulin synthesis begins in the β-cell with preproinsulin, which is converted to proinsulin after cleavage of the signal peptide. Within secretory vesicles, proinsulin is processed by prohormone convertases and carboxypeptidase E into insulin and C-peptide (Figure 1) [27,28]. Under physiological conditions this conversion is highly efficient [39]. Increased secretion of intact proinsulin can occur when secretory demand exceeds processing capacity or when β-cell function is impaired. In this sense, elevated iPI reflects the quality of hormone processing and contributes to the assessment of functional β-cell mass [9,29,30].

An elevation of intact proinsulin in peripheral blood is now regarded as an early and specific marker of impaired beta-cell function. Particularly informative in this context is the proinsulin-to-insulin ratio (PI/I), which reflects the efficiency of hormonal maturation within the beta cell [11,34,39].

Studies show that elevated intact proinsulin levels or an increased proinsulin-to-insulin ratio (PI/I):

- Are often already pathological in normoglycemic metabolic states [2,9,10];
- Are associated with an impaired first phase of insulin secretion [11,26];
- May indicate altered granule formation and secretory stress in the beta cell [40,41];
- Are linked to an increased risk of progression to overt glucose intolerance [36,42,43].

The diagnostic value of intact proinsulin lies in its potential to detect impaired processing before fasting glucose or HbA1c becomes abnormal. Sampling time is important. Fasting iPI predominantly reflects basal secretory demand and chronic processing stress and is readily standardized. Stimulated iPI, measured after a standardized glucose challenge, assesses processing capacity under metabolic stress and may reveal abnormalities that are not apparent in the fasting state. During an OGTT, measurements at baseline and at defined post-load time points—commonly 30, 60, and/or 120 min—allow evaluation of the magnitude and persistence of the proinsulin response. Existing studies have used different protocols; therefore, the optimal time point and clinically validated cut-off for normoglycemic individuals remain to be established prospectively [36,42,43].

Laboratory interpretation requires careful attention to assay methodology. Assays described as “total proinsulin” may detect intact proinsulin together with partially processed intermediates, whereas intact-proinsulin assays are designed to preferentially recognize the uncleaved molecule. Available methods differ in antibody epitopes, cross-reactivity with split proinsulins, calibration, analytical sensitivity, sample matrix, preanalytical stability, and reference intervals. Accordingly, absolute values and cut-offs cannot be assumed to be interchangeable between methods. The TECO^®^ Human Intact Proinsulin ELISA (TE1012; TECOmedical, Sissach, Switzerland) is one example used in several studies contributing to the present concept [10,34,42]. This assay is a quantitative two-site sandwich ELISA for intact human proinsulin in serum or EDTA/heparin plasma, requiring 50 µL sample volume, with a measuring range of approximately 3–100 pmol/L and analytical sensitivity of 0.15 pmol/L. Two monoclonal antibodies are directed against epitopes at the C-peptide/insulin A-chain and insulin β-chain/C-peptide junctions, thereby selectively detecting intact proinsulin. The cut-off for elevated intact proinsulin levels is 7 pmol/L and the intact proinsulin standard provided with the test kit is calibrated against the WHO 09/296 international standard. However, the concept is not dependent on this specific commercial assay. Any assay used for clinical or research classification should document analytical specificity for intact proinsulin and should be used consistently during longitudinal follow-up; assay-specific cut-offs require independent validation.

In addition to its role as a biomarker, intact proinsulin has been investigated for biological effects that differ from those of mature insulin [44,45,46,47]. These observations are of mechanistic interest but do not establish intact proinsulin as a causal mediator of diabetic complications. In the present concept, its primary role is therefore that of a marker of impaired proinsulin processing and β-cell secretory stress.

Studies providing prospective evidence that proinsulin abnormalities precede the development of type 2 diabetes in individuals who are normoglycemic at baseline are listed in Table 1.

## 6. Clinical Picture of Normoglycemic Diabetes

The clinical presentation of “normoglycemic diabetes” is characterized by a paradox: despite normal glucose levels and a normal HbA1c, structural and functional changes are already present that are typically seen in manifest diabetes mellitus—particularly impaired β-cell function, early endothelial damage, and the occurrence of diabetes-typical complications. This condition escapes conventional diagnostics, because fasting blood glucose, HbA1c, and often even a standardized OGTT do not yield abnormal results. Only more differentiated functional tests, such as insulin and proinsulin measurements, reveal that endocrine pancreatic function is already substantially impaired. Normoglycemic diabetes therefore represents a preclinical but clinically relevant entity that, in routine practice, has so far largely been overlooked [4,42].

At the centre of normoglycemic diabetes is the recognition that normal blood glucose levels do not guarantee metabolic health. Patients with markedly impaired β-cell function—due, for example, to genetic predisposition, chronic overload, or epigenetic programming—can maintain their glucose levels through compensatory hyperinsulinemia. This leads to a state of “masked β-cell insufficiency”: glucose levels remain within the normal range, but only at the cost of pathological (pro)insulin regulation [10].

Typical laboratory findings at this stage may include elevated intact proinsulin despite normal glucose values, a delayed or quantitatively inappropriate insulin response during OGTT, and reduced first-phase insulin secretion. In some research settings, reduced anatomical β-cell mass has also been demonstrated, but this cannot currently be assessed routinely. These findings are therefore best interpreted as evidence of impaired functional β-cell mass rather than as proof of structural β-cell loss in an individual patient [9,10,11,42,54,55].

Normoglycemic diabetes occurs preferentially in specific constellations in which β-cell stress or genetic vulnerability is present. Typical risk groups include individuals with a family history of type 2 diabetes or impaired glucose metabolism; lean, metabolically vulnerable individuals with β-cell-specific genetic susceptibility, for example TCF7L2; patients with metabolic dysfunction-associated steatotic liver disease (MASLD) despite normal HbA1c; women with a history of gestational diabetes; patients with polyendocrine metabolic ovarian syndrome (PMOS; formerly polycystic ovary syndrome, PCOS); individuals with an abnormal OGTT pattern, such as “late dumping”; and prepubertal children with rapid weight gain [56,57,58].

These groups benefit particularly from differentiated diagnostics, because conventional screening strategies such as HbA1c and fasting glucose do not capture their metabolic disorder. A particularly concerning feature of normoglycemic diabetes is the occurrence of diabetes-typical complications in the absence of hyperglycemia. Various studies have reported retinal microaneurysms or early vascular changes, increased hepatic fat content (MASLD), endothelial dysfunction and reduced NO release, subclinical neuropathic changes such as reduced nerve conduction velocity, and enhanced thrombotic tendencies [59,60,61].

These findings support the hypothesis that the pathophysiological consequences of β-cell dysfunction are not primarily mediated by hyperglycemia, but rather by secretory malfunction products such as intact proinsulin, chronically elevated insulin levels, inflammatory signals, and metabolic dysregulation.

## 7. Potential Parallel Development of Diabetes-Related Complications

Of particular importance in the context of normoglycemic diabetes is the observation that typical diabetes-related complications—such as microangiopathic changes (e.g., retinopathy, microalbuminuria, and polyneuropathy) or endothelial dysfunction—may already occur despite normal glucose levels. When loss of pulsatility is present for sufficiently long time microcirculation disorders may occur independent from the quality of glucose control. This suggests that the traditional definition of diabetes, centered on hyperglycemia as the key criterion, is insufficient to adequately capture the risk of diabetes-related end-organ damage. Rather, functional parameters of beta-cell health—particularly proinsulin concentration—should be incorporated as complementary factors into risk assessment [37,38]. In addition to its role as a diagnostic biomarker, intact proinsulin is also gaining pathogenetic relevance. Experimental studies suggest that proinsulin may exert biological effects of its own that differ substantially from those of mature insulin [44,45]. These include activation of proinflammatory cytokine pathways, endothelial dysfunction with reduced nitric oxide release, and stimulation of adipogenesis—and thereby of visceral hormonal secretion—suggesting a possible contribution to vascular and neuronal complications [46,47]. These effects could help explain why diabetes-related complications, such as macroangiopathy and atherosclerosis, microangiopathy, MASLD, retinopathy, and polyneuropathy, may occur in some patients even before manifest hyperglycemia develops—namely as a consequence of increased proinsulin exposure and loss of insulin pulsatility. Evidence from clinical trials investigating the predictive value of elevated (intact) proinsulin levels for the occurrence of macrovascular events in healthy “non-diabetic” individuals is provided in Table 2.

The clinical picture of normoglycemic diabetes has immediate implications for prevention and care. It requires expanded diagnostics that go beyond glucose parameters, particularly proinsulin measurements and OGTTs with multiple time points. It defines new target groups for preventive interventions before hyperglycemia develops. It also raises the question of early therapeutic action, for example through lifestyle modification, incretin therapy, or early insulin sensitizers—not to lower blood glucose, but to protect the β-cell and the vasculature [10,77].

## 8. Diagnostic Perspectives

The identification of normoglycemic diabetes requires a diagnostic perspective that extends beyond glucose thresholds and directly assesses β-cell function. In the present proposal, intact proinsulin and proinsulin-related measures are considered candidate markers of impaired hormone processing and secretory stress. Insulin resistance, inflammation, adiposity, and other metabolic abnormalities may modify β-cell workload, but nonspecific biomarkers of these processes are not part of the defining criteria of normoglycemic diabetes.

As outlined above, intact proinsulin is a highly sensitive marker of inefficient hormonal processing within the β-cell. Particularly when assessed in relation to insulin levels, using the PI/I ratio, its measurement enables the early identification of secretory dysregulation, differentiation between compensated insulin resistance and primary β-cell weakness, and risk stratification even when glucose values remain within the normal range. A dynamic OGTT with proinsulin and insulin measurements at multiple time points, for example at 0, 30, 60, and 120 min, allows for an even more differentiated functional assessment [10,34,42] (see Figure 2).

## 9. Therapeutic Implications

The concept of normoglycemic diabetes shifts the therapeutic focus away from glucose lowering alone and toward preservation of functional β-cell mass and prevention of vascular complications before glycemic deterioration occurs. The central clinical question is whether individuals with objectively demonstrated β-cell dysfunction during normoglycemia benefit from targeted intervention. At present, lifestyle measures remain the established preventive approach, whereas pharmacological treatment specifically for “normoglycemic diabetes” has not been validated in prospective outcome trials.

The proposed therapeutic goal in normoglycemic diabetes is preservation of functional β-cell mass and prevention or delay of progression to dysglycemia and/or vascular complications. This is conceptually different from treating an elevated glucose value: intervention is directed at reducing β-cell workload and preserving secretory competence before conventional diagnostic thresholds for prediabetes or diabetes are crossed.

Even in the normoglycemic stage, lifestyle modification remains the most important and effective intervention—although with a different therapeutic focus than in manifest diabetes: weight reduction, particularly in the presence of visceral obesity, reduces insulin resistance and inflammatory stress, while regular physical activity improves peripheral glucose utilization and lowers insulin requirements, thereby directly relieving the β-cell [78,79,80,81].

Dietary quality, adequate sleep, weight management where appropriate, and regular physical activity can reduce metabolic demand and insulin resistance, thereby lowering β-cell workload and vascular risk. These measures should be understood as established components of diabetes prevention rather than as treatments validated specifically for the proposed entity of normoglycemic diabetes [78,79,80,81,82,83,84,85].

Pharmacological therapy specifically for normoglycemic diabetes has not been established. Although several glucose-lowering drug classes can reduce β-cell workload or modify insulin demand in established diabetes, their use in normoglycemic individuals solely on the basis of a proinsulin abnormality requires prospective evidence of clinical benefit and an acceptable benefit-risk balance.

## 10. New Strategies: β-Cell-Restorative Approaches

In early stages of β-cell dysfunction, some components of impaired secretory function may remain reversible. This provides a rationale for research into interventions designed to reduce β-cell workload and preserve functional β-cell mass. However, strategies intended specifically to restore β-cell mass, improve prohormone processing, or normalize secretory dynamics remain investigational in normoglycemic individuals and require prospective validation.

If future intervention trials are conducted in normoglycemic individuals with documented β-cell dysfunction, treatment response should be assessed using prespecified functional endpoints. Intact proinsulin or proinsulin-related ratios may be candidate biomarkers, but assay method, fasting versus stimulated sampling, and clinically meaningful change thresholds must first be standardized and validated.

The therapeutic implication of the concept is therefore preventive rather than glucose-lowering: detection of impaired functional β-cell mass may define a biologically enriched group in whom established preventive measures can be intensified and future β-cell-preserving strategies can be studied. The decisive research question is whether intervention based on β-cell functional markers improves outcomes beyond conventional risk-based prevention.

## 11. Discussion

Normoglycemic diabetes is proposed as a pathophysiological disease concept intended to make early impairment of functional β-cell mass and elevated macrovascular risk visible before hyperglycemia occurs. At its core are abnormalities of insulin secretion and proinsulin processing that can, in principle, be measurable while conventional glucose criteria remain normal. The concept should be regarded as hypothesis-generating until prospective studies establish reproducible diagnostic criteria and demonstrate clinical utility.

This proposal differs from conventional diabetes prevention. Prevention identifies individuals at increased future risk and applies interventions intended to reduce that risk; normoglycemic diabetes, by contrast, is intended to designate measurable β-cell pathology already present during normoglycemia and consecutively increased vascular risk. The proposed sequence is therefore conceptual: preserved functional β-cell mass → normoglycemic β-cell dysfunction → glucose-defined prediabetes → overt type 2 diabetes. Recognition of an earlier pathological state may inform prevention, but it is not synonymous with prevention.

If validated, the concept could expand early diagnostics by adding functional β-cell assessment to conventional glucose-based classification. Intact proinsulin is emphasized as a candidate marker because it reflects impaired prohormone processing and β-cell secretory stress. Importantly, intact proinsulin measurement can be standardized against the WHO International Standard 09/296, and a fasting cut-off of 7 pmol/L has been established for the identification of pathological elevations when using appropriately validated specific intact proinsulin assays [34,77]. Nevertheless, assay characteristics and analytical comparability should be considered when applying this threshold across different platforms. The principal therapeutic implication would be earlier identification of β-cell stress and the opportunity to test whether reducing β-cell workload preserves functional β-cell mass, reduces macrovascular risk and delays progression to dysglycemia and overt type 2 diabetes.

Important questions remain open. What proportion of normoglycemic individuals with abnormal proinsulin processing progresses to glucose-defined prediabetes or type 2 diabetes? Which measure best reflects declining functional β-cell mass: fasting intact proinsulin, stimulated intact proinsulin, PI/I, PI/C-peptide, or a composite functional index? Which sampling time and assay-specific threshold provides the best discrimination? To what extent do functional abnormalities reflect reversible β-cell stress versus loss of anatomical β-cell mass? Most importantly, does marker-guided intervention improve patient-relevant outcomes beyond established risk-factor-based prevention?

Current classification systems, such as those of the WHO and ADA, define prediabetes and diabetes exclusively by glucose thresholds, including fasting glucose, OGTT, and HbA1c. The concept of normoglycemic diabetes challenges this classification—not in order to replace it, but to complement it. The ADA Standards of Care 2026 continue to diagnose prediabetes and diabetes using glycaemic criteria (fasting plasma glucose, 2 h OGTT glucose and HbA1c), while defining type 2 diabetes pathophysiologically as a progressive loss of adequate β-cell insulin secretion, frequently on the background of insulin resistance [1]. Thus, the proposed model does not dispute established ADA diagnostic thresholds; rather, it addresses a potentially earlier biological stage in which functional β-cell abnormalities may already be measurable while all conventional glycaemic criteria remain normal. In this sense, normoglycemic diabetes is proposed as a complementary mechanistic layer, not as a replacement for ADA classification.

The concept is also aligned with the ADA/EASD Precision Medicine in Diabetes Initiative, which emphasizes heterogeneity in diabetes aetiology, pathogenesis, prognosis and treatment response and calls for biologically informed precision diagnosis and prevention [86]. Importantly, the 2023 international consensus report also stresses that biomarker-defined subgroups require rigorous prospective validation before routine clinical implementation. We therefore regard normoglycemic diabetes as a candidate β-cell-centred precision-diagnostic phenotype rather than an established clinical diagnosis.

Recent concepts of preclinical type 2 diabetes likewise distinguish glycaemic stage from underlying biological disease stage. Pathophysiology-based subphenotyping has demonstrated that individuals at increased risk can already be separated into metabolically distinct clusters before overt diabetes, with different trajectories for progression and complications [87]. More recent work using continuous glucose monitoring and data-driven metabolic phenotyping further supports substantial heterogeneity before conventional diagnostic thresholds are crossed [86,88]. These findings support the general principle that a glucose-only classification does not fully capture early disease biology, while they do not by themselves validate intact proinsulin or the term “normoglycemic diabetes” as a diagnostic entity.

## 12. Future Perspectives

The continued glucose-centred definition of prediabetes is becoming increasingly difficult to defend in light of recent evidence. While prediabetes is still formally defined by glycaemic thresholds, this operational classification appears to lag behind the underlying biology. Recent studies have shown that marked metabolic heterogeneity, abnormal glycaemic response patterns and distinct pathophysiological subphenotypes can already be detected before overt diabetes is diagnosed and are not adequately reflected by fasting glucose, HbA1c or standard OGTT cut-offs [88,89]. In particular, emerging data indicate that impaired β-cell function is not simply a late consequence of dysglycaemia, but represents an early and central defect in disease evolution [90]. From this perspective, the current definition of prediabetes is not wrong, but biologically incomplete: it identifies the stage at which dysglycaemia becomes measurable, not the stage at which diabetic disease actually begins. This has important clinical implications, because a purely glucose-based framework risks missing individuals who are still classified as normoglycaemic despite already having clinically relevant β-cell dysfunction and possibly early vascular injury. We therefore argue that the existing concept of prediabetes should be expanded by a mechanistic dimension that incorporates early β-cell failure as a defining element of metabolic risk. In this context, the concept of normoglycemic diabetes is not intended to replace established diagnostic categories, but to address a pathophysiological blind spot in current diabetology: the presence of a clinically relevant diabetic disease process before the onset of diagnostic hyperglycaemia. In interpreting this evidence, we distinguish evidence-based observations (early β-cell dysfunction and metabolic heterogeneity before diabetes) from the hypothesis advanced here (that a reproducible biomarker-defined normoglycemic state should be classified as a distinct disease concept).

Differentiated diagnostics of normoglycemic diabetes opens the prospect of marker-guided prevention based on β-cell biology rather than generalized risk alone. In this framework, intact proinsulin and dynamic insulin/proinsulin responses would be evaluated together with established clinical risk factors. The focus would then no longer be exclusively on hyperglycemia, but on the earlier detectable decline in functional β-cell mass. This proposal is consistent with previous β-cell-centric frameworks emphasizing reduction in β-cell workload and preservation of functional β-cell mass as central objectives in the prevention and management of type 2 diabetes [90,91,92]. The specific contribution of the present concept is to ask whether objectively demonstrable β-cell dysfunction during normal glucose tolerance should itself be recognized as a clinically meaningful early disease state.

The recognition and classification of normoglycemic diabetes opens up decisive clinical and preventive opportunities:

- Early diagnosis: identification of at-risk individuals before irreversible β-cell damage and clinically manifest hyperglycemia occur.
- Differentiated risk assessment: integration of β-cell functional measures, particularly intact proinsulin and standardized dynamic testing, with established clinical risk factors.
- Targeted early intervention: established lifestyle-based preventive measures, with pharmacological β-cell-preserving strategies reserved for prospective evaluation until clinical benefit is demonstrated.
- Prevention of diabetic complications: through timely intervention in the functional early stage.

In the long term, the proposed concept of normoglycemic diabetes may provide a mechanistic bridge between apparently normal glucose regulation and glucose-defined prediabetes. At present, however, it should be regarded as a testable disease hypothesis rather than an established diagnostic category. Its clinical value will depend on prospective demonstration that β-cell functional markers identify a reproducible high-risk state and that acting on this information improves outcomes beyond current prevention strategies.

The present work is intended as an impulse, a foundation, and an appeal to scientific colleagues to initiate studies that support the further development of diagnostic algorithms, clinical guidelines, and pathophysiological models—approaches that better reflect the reality of the early phase of diabetes, long before glucose levels deteriorate.

## Figures and Tables

**Figure 1:**
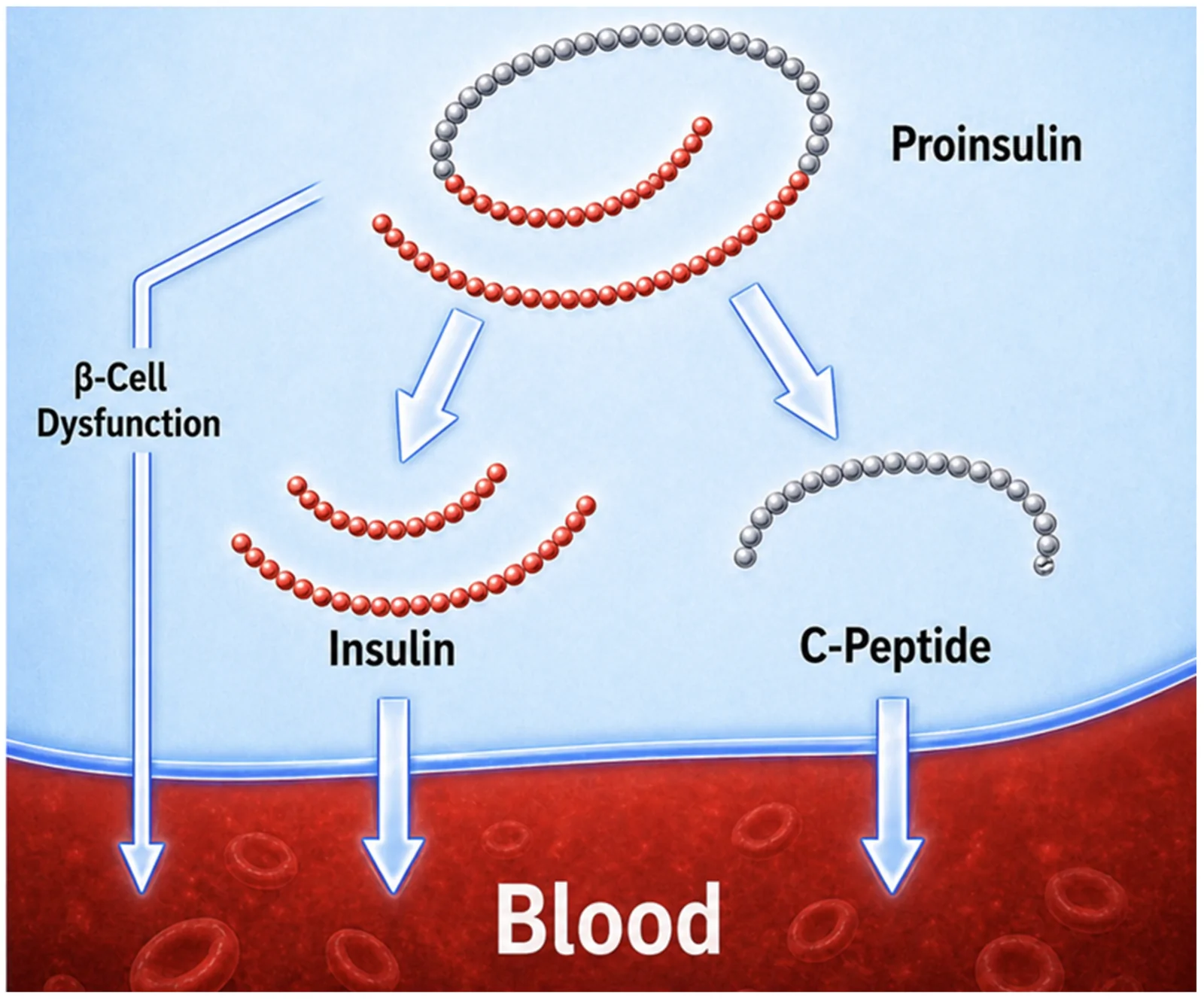
Cleavage of intact proinsulin in the ß-cells under normal and pathologic conditions. The biochemical cleavage of proinsulin to insulin and C-peptide is established physiology. Increased release of incompletely processed proinsulin during β-cell secretory stress is supported by experimental and clinical evidence. The use as a defining marker of the proposed entity “normoglycemic diabetes” remains hypothesis-generating and requires prospective validation.

**Figure 2:**
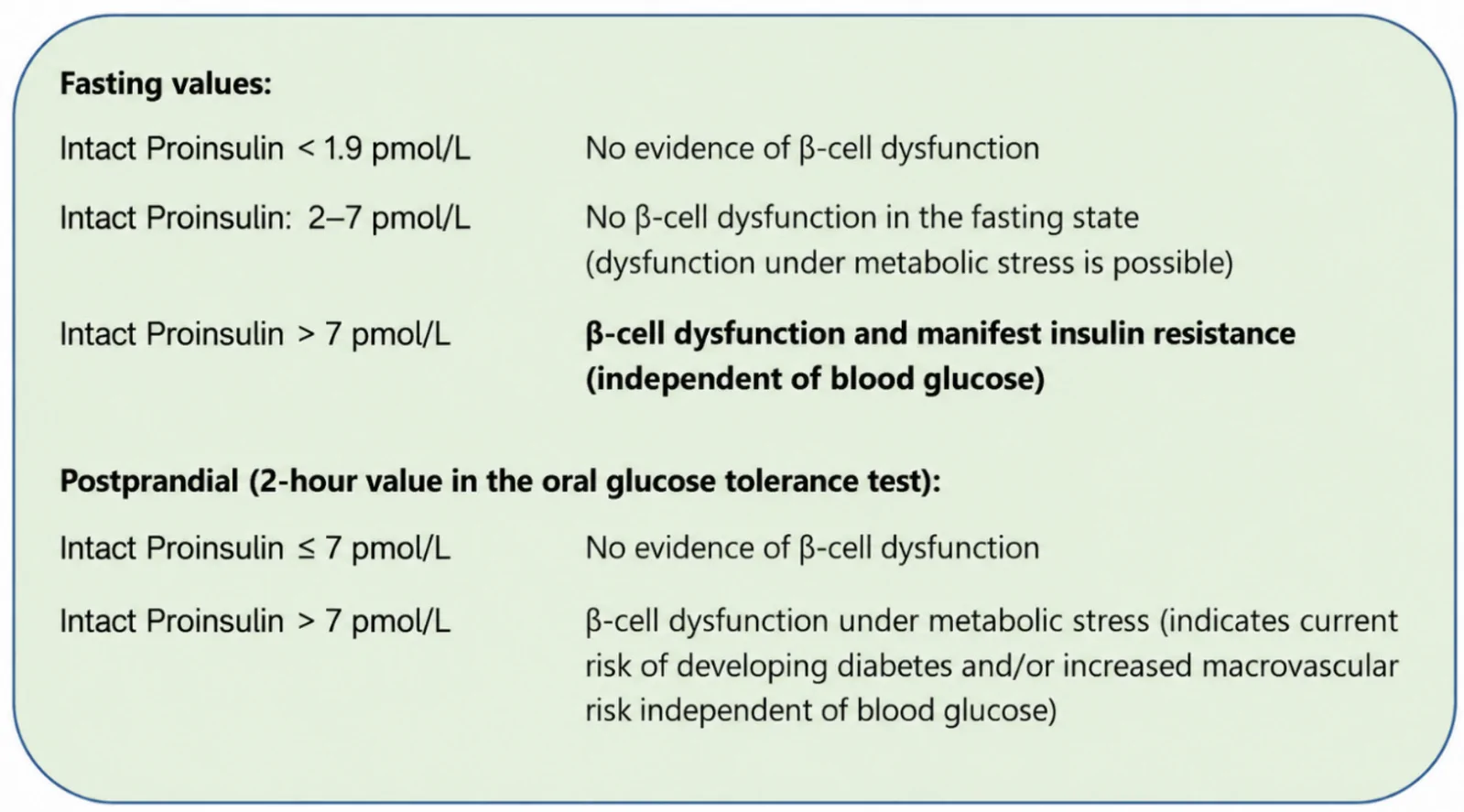
Diagnostic interpretation of intact proinsulin levels in the presence of normal blood glucose and HbA1c values. Note: numerical thresholds shown in this figure are assay-specific examples and should not be interpreted as universal diagnostic cut-offs. Fasting and post-load values must be interpreted according to the analytical method and validated sampling protocol used. The 7 pmol/L threshold for elevated intact proinsulin is based on specific assays calibrated against the WHO International Standard 09/296. The interpretation of an elevated value during otherwise normal glucose regulation as evidence supporting the proposed entity “normoglycemic diabetes” is hypothetical and requires prospective clinical validation. Post-load interpretation additionally depends on a standardized sampling protocol.

**Table 1:** Prospective evidence relating proinsulin measures to subsequent type 2 diabetes in populations/subgroups with normal glucose tolerance (NGT) at baseline. Effect estimates are shown only when reported and directly applicable. “Evidence type” is a qualitative description of study design and methodological strength and is not a formal GRADE or ADA evidence grade.

Reference	Study Characteristics	Core Results	Adjusted Effect Estimate (When Available)	Major Limitations	Evidence Type
Mykkänen et al., 1995 [48]	Prospective Cohort study with matched-pair analysis; N = 893; 3.5 years	A disproportionate increase in proinsulin compared with insulin predicts the development of type 2 diabetes.	RR 1.37 per 1-SD increase in PI/insulin ratio (*p* = 0.020), independent of fasting glucose.	Elderly Finnish cohort; matched case–control analysis nested in cohort; mixed NGT/IGT baseline; older proinsulin assays.	Prospective observational; moderate supportive evidence
Kahn et al., 1995 Kahn et al., 1996 [49,50]	Prospective controlled comparative study; N = 87; 5 years	Elevated proinsulin levels reliably predict the development of T2DM in Japanese-American men with NGT/IGT.	Relative odds 2.17 for fasting PI after adjustment for fasting insulin, intra-abdominal fat and BMI (reported in 1995 analysis).	Small sample (N = 87); men only; Japanese-American population; mixed NGT/IGT baseline; limited event number.	Prospective observational; moderate supportive evidence
Haffner et al., 1997 [6]	Prospective Cohort study; N = 2282, including 1449 NGT subjects; 3.25 years	Fasting PI and the PI/INS ratio reliably predict the development of T2DM, whereas insulin does not.	Adjusted association reported; exact adjusted OR not consistently provided in accessible summary.	Nested case–control analysis; mixed NGT/IGT; total immunoreactive proinsulin rather than intact PI; short follow-up.	Prospective observational; moderate supportive evidence
Wareham et al., 1999 [36]	Prospective cohort; NGT subgroup N = 1041; 4.5 years	Within the NGT subgroup, elevated fasting 32,33 split-proinsulin predicted subsequent T2DM.	RR top vs. bottom quartile: 32,33 split PI 16.4 (95% CI 2.2–121.9); intact PI 5.2 (1.5–17.3), independent of age, sex, BMI and baseline glucose tolerance.	Only 26 incident diabetes cases; wide confidence intervals; split-proinsulin assay; cohort included non-diabetic participants beyond strict NGT.	Prospective population cohort; moderate supportive evidence
Zethelius et al., 2003 [51]	Prospective Cohort study N = 1000, 27 years	Elevated iPI is predictive of T2DM in Swedish men.	Adjusted estimate not reported in the manuscript summary used here.	Men only; historical cohort; assay and diagnostic criteria predate current standards; NGT status not uniformly specified for all analyses.	Prospective cohort; supportive evidence
González-Sánchez et al., 2008 [52]	Prospective cohort; N = 1946, all with NGT at baseline; 6 years	Elevated fasting and post-load proinsulin measures were associated with subsequent development of T2DM.	Adjusted estimate not reported in the manuscript summary used here.	Population-specific cohort; assay/platform heterogeneity; residual confounding possible despite prospective design.	Prospective cohort; supportive evidence
Pfützner et al., 2015 [53]	Prospective Cohort, NGT N = 23, 5 years	Elevated iPI during OGTT, particularly the 2 h value, is predictive of T2DM.	Adjusted estimate not reported in the manuscript summary used here.	Very small exploratory cohort (N = 23); limited number of converters; limited generalizability.	Small prospective cohort; exploratory evidence
Vangipurapu et al., 2015 [43]	Prospective Cohort study N = 9306, 6 years	Fasting and postprandial iPI are highly predictive of T2DM in Finnish adults.	Fasting PI HR 1.46 (95% CI 1.41–1.52); 120 min PI HR 1.44 (1.38–1.50), adjusted for age, BMI, smoking, physical activity and follow-up time.	Men only; Finnish population; participants were non-diabetic rather than exclusively NGT; associations attenuated after adjustment for insulin sensitivity/secretion.	Large prospective population cohort; relatively strong observational evidence

Abbreviations: NGT = normal glucose tolerance; IGT = impaired glucose tolerance; iPI = intact proinsulin; T2DM = type 2 diabetes mellitus; OGTT = oral glucose tolerance test.

**Table 2:** Studies confirming the predictive value of (intact) proinsulin as biomarker for macrovascular events in non-diabetic subjects. Effect estimates are shown only when reported and directly applicable. Evidence categories are qualitative descriptions of study design and do not imply a formal GRADE or ADA recommendation level.

Reference	Study Characteristics	Core Result	Adjusted Effect Estimate (When Available)	Major Limitations	Evidence Type
Båvenholm et al., 1995 [62]	Case–Control Study N = 140, cross-sectional	In young men with NGT, elevated fasting iPI is a predictor of CHD.	No adjusted HR/OR reported; multivariable regression identified basal proinsulin as an independent correlate of coronary atherosclerosis.	Cross-sectional case–control design; young male MI survivors; association does not establish prospective prediction or causality.	Cross-sectional observational; low-to-moderate evidence
Katz et al., 1996 [63]	Case–Control Study N = 116, cross-sectional	PI is an early CHD risk marker in individuals with NGT.	Not reported in accessible summary.	Case–control/cross-sectional design; small sample; residual confounding; older assay methodology.	Cross-sectional observational; low evidence
Yudkin et al., 1997 [64]	Prospective cohort study; N = 1794 (1139 Caucasian, 655 African-origin participants); 5 years	In both ethnic groups, PI is a more robust marker of prevalent and incident CHD than INS.	Not reported in accessible summary.	Ethnic heterogeneity; observational design; proinsulin-like molecules rather than standardized intact PI in all analyses.	Prospective cohort; supportive evidence
Haffner et al., 1998 [65]	Cross-sectional study; N = 1070; diverse ethnic groups	PI is associated with increased carotid intima-media thickness and therefore proved to be a marker of subclinical atherosclerosis.	No hazard ratio; association based on multivariable analysis of carotid intima-media thickness.	Cross-sectional surrogate endpoint; cannot establish incident events or causality.	Cross-sectional observational; low evidence
Lindahl et al., 1999 [66]	Prospective Case–Control Study N = 216, 8 years	In individuals with NGT, PI predicts the risk of a first myocardial infarction independently of other risk factors.	Highest vs. lowest proinsulin quartile: OR 3.5 (95% CI 1.2–9.9) for first AMI; association persisted after several cardiovascular adjustments but attenuated after BMI adjustment.	Incident case–control design; modest sample; BMI materially influenced the association; non-randomized.	Prospective nested case–control; moderate supportive evidence
Lindahl et al., 2000 [67]	Prospective Case–Control Study N = 225, 8 years	In individuals with NGT, PI predicts the risk of stroke independently of other risk factors, with a stronger association in women.	Adjusted estimate not reported in accessible summary.	Prospective case–control design; relatively small sample; potential sex interaction; observational.	Prospective nested case–control; moderate supportive evidence
Yudkin et al., 2002 [68]	Prospective Cohort study N = 1181, 10–14 years	Fasting PI predicts CHD in men independently of INS and conventional risk factors.	Standardized OR 1.54 (95% CI 1.07–2.20) for sum of proinsulin-like molecules after age/BMI adjustment; attenuated after additional cardiovascular risk-factor adjustment.	Men only; proinsulin-like molecules rather than standardized intact PI; attenuation with confounder adjustment.	Prospective cohort; moderate supportive evidence
Zethelius et al., 2002 [69]	Prospective Cohort study N = 1142, 27 years	iPI is an independent predictor of coronary heart disease in non-diabetic 50-year-old men.	Adjusted estimate not reported in accessible summary.	Men only; age-specific Swedish cohort; historical assay; observational design.	Long-term prospective cohort; supportive evidence
Oh et al., 2002 [70]	Prospective Cohort study N = 1127, 8 years	PI is an independent predictor of CHD in older adults with NGT, with a stronger association in men.	Adjusted estimate not reported in accessible summary.	Older adults; cohort-specific demographics; observational design; residual confounding.	Prospective cohort; supportive evidence
Juhan-Vague et al., 2003 [71]	Case–control study; N = 1251; cross-sectional	PI is a significant risk factor for myocardial infarction in men with the PAI-1 −675 4G/4G genotype.	Not reported/not applicable for isolated proinsulin effect.	Genotype-specific subgroup analysis; case–control design; limited generalizability.	Case–control observational; low evidence
Dunder et al., 2004 [72]	Prospective Cohort study N = 1000, 9 years	Proinsulin and the ApoB/ApoA1 ratio predict acute coronary events in middle-aged men.	Composite ULSAM score including proinsulin and ApoB/ApoA1: HR 1.77 per 1-SD increase (95% CI 1.49–2.10).	Effect estimate pertains to a composite score, not proinsulin alone; men only; derivation/validation within one cohort.	Prospective cohort; supportive evidence for multivariable risk model
Zethelius et al., 2005 [73]	Prospective Cohort study N = 1064, 10 years	iPI predicts CHD, whereas INS is not predictive.	Intact proinsulin HR 1.18 (95% CI 1.10–1.38) for CHD in reported analysis.	Men aged ~70 years; limited generalizability; observational; residual confounding possible.	Prospective cohort; moderate supportive evidence
Alssema et al., 2005 [74]	Prospective Cohort study N = 632, 11 years	iPI is an independent predictor of all-cause and cardiovascular mortality.	Adjusted estimate not reported in accessible summary.	Moderate sample; observational; mortality influenced by multiple competing risks.	Prospective cohort; supportive evidence
Kronborg et al., 2007 [75]	Prospective Cohort study N = 1125, 7 years	The PI/INS ratio predicts progression of carotid plaque formation, especially in women.	Adjusted estimate not reported in accessible summary.	Surrogate endpoint (plaque progression); observational; sex-specific effects.	Prospective cohort; supportive evidence
Wiberg et al., 2009 [76]	Prospective Cohort study N = 1016, 8.8 years	iPI predicts stroke, whereas INS does not.	Adjusted estimate not reported in accessible summary.	Older male cohort; observational; endpoint-specific; assay comparability limitations.	Prospective cohort; supportive evidence

NGT = Normal glucose tolerance; (i)PI = (Intact) Proinsulin; Abs = Antibodies; pp = postprandial; INS = Insulin, CHD = coronary heart disease.

## Data Availability

No new data were created or analyzed in this study. Data sharing is not applicable to this article.
